# Chat-GPT: Opportunities and Challenges in Child Mental Healthcare

**DOI:** 10.12669/pjms.39.4.8118

**Published:** 2023

**Authors:** Nazish Imran, Aateqa Hashmi, Ahad Imran

**Affiliations:** 1 Dr. Nazish Imran, MBBS; FRCPsych(London); MRCPsych (London); MHPE;PhD. Professor, Department of Child & Family Psychiatry, King Edward Medical University/Mayo Hospital, Lahore, Pakistan; 2 Dr. Aateqa Hashmi, MBBS. House Officer, Department of Child & Family Psychiatry, King Edward Medical University/Mayo Hospital, Lahore, Pakistan; 3 Ahad Imran, Third year Computer Sciences Student, Syed Babar Ali School of Science & Engineering (SBASSE), Lahore University of Management Sciences (LUMS), Lahore, Pakistan

**Keywords:** Artificial Intelligence, Child Mental Health, ChatGPT, Open AI, Mental Illness

## Abstract

Mental health in children and young people is a global public health concern. With the increasing prevalence of mental illnesses and a significant treatment gap, Mental Health in children and adolescents is now a global public health concern. The development and extensive research in the field of Artificial Intelligence(AI) for healthcare has been quite promising. The emergence of AI based alternatives could be a viable solution for reducing mental health treatment gap for people belonging to low and middle income countries. Development of Chatbots like ChatGPT which is trained, using large amount of textual data from the internet, can revolutionize child and adolescent mental healthcare by acting as an effective assisting tool but a lot of caution is required for its safe and responsible use in times to come.e

“ChatGPT puts the power of AI in everyone’s hand.”

*- Fredrick Godin*

ChatGPT is making a significant impact in everyone’s life around the globe, including younger population. More than 25% of the population of the world is under the age of 14. Mental Health in children and adolescents is a pressing public health issue. The World Health Organization estimates that nearly 20% of these children suffer from psychiatric problems.^1^ With huge treatment gap in Child and adolescent mental health care especially in low and middle-income countries and increase need for psychiatric diagnosis and treatment services, artificial intelligence (AI) and digital interfaces are emerging as viable alternatives for reducing this gap. The ChatGPT (Chat-Generative Pre-Trained Transformer) and other AI-based chatbot’s ability to generate human-quality responses can help in making mental health services accessible and affordable.^2^

Chat-GPT is an AI-based language model which uses natural language processing (NLP) and conversational AI to help mental health professionals deliver more personalized and effective mental health support. This is possible because the system is powered by a machine learning algorithm, trained using large dataset of clinical interactions in healthcare. ChatGPT can support child & adolescent mental health by supporting physicians as well as patients and families in various ways.

## Benefits for Mental Health Practitioners:

## A resource for Mental health clinicians:

ChatGPT could be used to analyze a child and adolescent’s language text for patterns and correlations that can reveal underlying mental health issues. It can help clinicians in providing quick access to assessment tools, new evidence-based treatment options and best clinical practices for managing common child & adolescent mental health problems. It can also flag potential drug interactions and help in reducing prescription errors, while saving clinician’s time.^3^

## Enhance Proactive treatment adjustments:

ChatGPT can assess intensity of negative cognitive distortions by analyzing patients’ language patterns in real time, which alongside clues from mental health state examination may help clinicians to improve diagnostic accuracy, determine early treatment response and enhance proactive adjustments in treatment.^4^,^5^

## Extracting Information from Large sets of Data:

Chat GPT can use its vast database of language to generate reports and summaries from large sets of data, such as patient interviews. This could assist mental health practitioners to diagnose children and adolescents and develop personalized treatments quickly and accurately. Lab reports, imaging results and any other relevant information can also be extracted quickly from patients records.^5^

## Facilitation of Communication:

ChatGPT can facilitate communication between young patients and mental health professionals by precise and swift translation of medical jargon and technical terminologies due to its advanced language processing capabilities. It thus allows clinicians to help engage children and adolescents in their management plan and better understand their diagnosis and treatment options.

## Integration of ChatGPT enabled apps in healthcare devices:

ChatGPT enabled apps and its potential integration in healthcare devices such as smart wearables, and other monitoring devices is one of the most exciting developments and can have a potential future role in offering real time insight in child & adolescent health status .

## Teaching and Training in child mental health:

ChatGPT can be effective tool in medical education, Child & adolescent mental health teaching and training by identifying gaps in knowledge, for content creation, producing high quality engaging content at scale, helping in lesson planning and assessments.^6^ This can be very cost effective in improving awareness of child and adolescent mental health amongst schoolteachers and allied pediatric health professionals.

## Reduction in Occupational stress:

By providing ‘short cuts’ for time consuming, but menial tasks, ChatGPT can help to extend child mental health professionals time and resources, and potentially reduce occupational stress.

## Benefits for Patients and Families:

## Easy access to information about Mental health issues:

Children and adolescents can better comprehend and look after their mental health by using ChatGPT to know more about mental illnesses, various coping methods, and treatment choices Easy access to mental health support:

Stigma, financial constraints, poor access to services and reluctance to seek traditional therapies due to cultural and societal myths are some of the barriers preventing children and adolescents from receiving mental healthcare they need.^7^ ChatGPT and AI apps can provide children and adolescents with a non-judgmental and accessible means for mental health care. A research showed “Woebot” to be effective in reducing depression among college students after just two weeks of therapy modeled after cognitive behavioral therapy and was considered as empathetic too.^8^ In another ongoing project, The WHO Sustainable Technology for Adolescents and youth to Reduce Stress (STARS) led to development of a chatbot that delivers transdiagnostic cognitive behavioral therapy (CBT) content. Conversational pattern in ChatGPT can mimic real time therapist especially for people with social anxiety disorder.

## Role in crisis:

ChatGPT can be used to triage patients by enquiring about their symptoms and relevant medical and past history to determine the severity and urgency of their condition. ChatGPT’s ability to provide 24/7 support ensuring constant availability of digital mental health can be particularly helpful for people in crisis. It can help control suicidal ideation or panic attacks when access to professional is not possible as they can get support from online apps. In case of suicidal thinking referenced directly in ChatGPT, it provides contact information for the Suicide and Crisis helplines.

## Help in Stress reduction:

ChatGPT can also be a practical tool for youngsters stressed with time management and organization. by breaking down complicated assignments into manageable steps. This can assist in managing anxiety linked with feeling overwhelmed.

## Limitation of Human Biases:

Risk of bias even subconsciously may occur during human interactions in therapy. ChatGPT may help in supporting mental health of children and adolescents, who may be concerned about their therapist judging them or who are unwilling to talk to a therapist about sensitive issues or sharing confidential information.

## Limitations:

Ben Buchanan and Andrew Imbrue argued in their Book “The New Fire War, Peace, and Democracy in the Age of AI” that

***“AI is like fire; if we control it, it can become a source of power; otherwise, it can become a source of destruction.”***

Despite the advantages of ChatGPT, it is unwise to dismiss the limitations and potential concerns regarding AI technologies.^5^,^6^ ChatGPT is trained on human-annotated data which is prone to bias. The model then identifies patterns in this data and subsequently may also shows bias in some scenarios. Although ChatGPT can provide evidence-based information, it cannot diagnose or advise a management plan exactly tailored to a child and adolescent’s specific needs. A lot of complex factors influencing child and adolescent mental health like socioeconomic status, educational and cultural factors, family dynamics cannot be addressed by ChatGPT. ChatGPT, if not prepared with data from authentic and reliable sites, may be potentially harmful to children and adolescents with psychiatric problems by providing inappropriate and wrong advice.

One of the main limitations is deficiency of human like understanding in AI technologies. In managing a child’s mental health conditions, therapeutic alliance between child and therapist is very important and is built through attunement, positive regard, and non-verbal communications, where ChatGPT falls short. It may not be sensitive to non-verbal communication or subtle signals of crisis by children and adolescents facing mental health issues like suicidal and homicidal risks. The inability to detect such warning signs and ask relevant questions to assess safety may lead to catastrophic outcomes. ChatGPT may not always identify when a child and adolescent require more assistance. Furthermore, Chatbots are also not capable of providing the same degree of emotional support as a trained therapist. However, they can be a helpful supplement to traditional therapy.^3^,^5^

Confidentiality and privacy are crucial factors in mental health support for children and adolescents, and it is essential to ensure that users’ information is kept secure. Any child or adolescent utilizing ChatGPT for their psychiatric issues is likely to share sensitive personal and family details, making them potentially vulnerable in cases of breach of confidentiality. American Psychiatric Association (APA) in view of the urgent need to regulate AI-based tools, has created a digital psychiatry task force to evaluate and monitor AI and mental health-related apps for their efficacy, safety, and potential to provide mental health care.

## CONCLUSION

To conclude, ChatGPT can revolutionize child and adolescent mental healthcare by acting as an effective assisting tool. But it would be unwise and impractical to suggest that it can replace human clinical judgment entirely. Safe and responsible usage is the key to make the best use of this assisting tool for mental health promotion in this vulnerable age groups.
